# Entropy Based Pythagorean Probabilistic Hesitant Fuzzy Decision Making Technique and Its Application for Fog-Haze Factor Assessment Problem

**DOI:** 10.3390/e22030318

**Published:** 2020-03-11

**Authors:** Bushra Batool, Mumtaz Ahmad, Saleem Abdullah, Shahzaib Ashraf, Ronnason Chinram

**Affiliations:** 1 Department of Mathematics, University of Sargodha, Sargodha 40100, Pakistan; bushra.batool@uos.edu.pk (B.B.); mumtaz.ahmad@uos.edu.pk (M.A.); 2 Department of Mathematics, Abdul Wali Khan University, Mardan 23200, Pakistan; saleemabdullah@awkum.edu.pk (S.A.); shahzaibashraf@awkum.edu.pk (S.A.); 3 Algebra and Applications Research Unit, Department of Mathematics and Statistics, Faculty of Science, Prince of Songkla University, Hat Yai, Songkhla 90110, Thailand

**Keywords:** Pythagorean probabilistic hesitant fuzzy set, extended TOPSIS technique, decision making problem

## Abstract

The Pythagorean probabilistic hesitant fuzzy set (PyPHFS) is an effective, generalized and powerful tool for expressing fuzzy information. It can cover more complex and more hesitant fuzzy evaluation information. Therefore, based on the advantages of PyPHFSs, this paper presents a new extended fuzzy TOPSIS method for dealing with uncertainty in the form of PyPHFS in real life problems. The paper is divided into three main parts. Firstly, the novel Pythagorean probabilistic hesitant fuzzy entropy measure is established using generalized distance measure under PyPHFS information to find out the unknown weights information of the attributes. The second part consists of the algorithm sets of the TOPSIS technique under PyPHFS environment, where the weights of criteria are completely unknown. Finally, in order to verify the efficiency and superiority of the proposed method, this paper applies some practical examples of the selection of the most critical fog-haze influence factor and makes a detailed comparison with other existing methods.

## 1. Introduction

In the 21st century, with the rapid economic and social development and the acceleration of industrialization, environmental problems, especially air pollution, have become increasingly severe restricting the economic development of many countries and threatening human health. The continued rise in fossil fuel consumption and emissions of contaminants releases a large amount of particulate matter (PM), which raises concentration of PM, reduces visibility and deteriorates air quality [1]. It is easy to cause fog-haze weather under certain meteorological conditions. Fog-haze is a serious threat to the health of people’s respiratory systems, and it is reported that more than 3 million people worldwide die each year from air pollution (mainly from PM) [2]. Hence, studying the issue of the key influence factor of fog-haze weather is meaningful. However, due to the rapid growth of the social economy and the limitation of experts’ professional knowledge and cognitive ability, it is somewhat challenging for a single expert to provide comprehensive evaluation information in the face of complex problems with fog-haze weather influencing factors. Thus, it is more reasonable that a group of experts use the comprehensive intelligence, cross-disciplinary knowledge and skills to evaluate the complex influence factors of fog-haze weather collaboratively.

Real life decision making problems are too complex and challenging due to the presence of different kinds of uncertainties and vagueness in the information data. In 1965, Zadeh [3] proposed the theory of fuzzy sets to model uncertain/vague information effectively. After the pioneering work of Zadeh [3], many generalizations and extensions of fuzzy sets (FSs) have been proposed by researchers and applied in a wide range of application areas. Intuitionistic fuzzy set (IFS), introduced by Atanassov [4], is one of the most significant extensions of the fuzzy set that has been extensively studied and implemented in different disciplines. Over the past thirty years, intuitionistic fuzzy set theory has been successfully employed to solve many problems connected to real life situations such as decision making [5].

Yager [6,7,8] models Pythagorean fuzzy set (PyFS) as a generalization of IFS, with the constraint that the square sum of positive and negative membership degrees is less than or equal to one. We can say that the space of all intuitionistic membership values is also Pythagorean membership values, but the converse is not true. For example, in an environment where the positive membership value is 0.6 and the negative membership value is 0.5, we cannot use IFSs because the sum of their membership values exceeds one. So, in this situation we use PyFSs to deal with the decision making problems. As a result, PyFS is stronger than IFSs to make a pact of ambiguity in daily life problems.

Frequently, hesitancy occurs everywhere in our universe. It is difficult to choose one of the best alternatives with the same features in real life. Due to vagueness and hesitancy in the data, experts are having complications in decision making. To overcome the hesitancy, Torra [9] proposed the notion of hesitant fuzzy set (HFS). After that, the enhanced form of hesitating fuzzy sets, the Pythagorean hesitant fuzzy set was proposed by Khan et al. [10]. Overhead notions can be used to determine randomness in an efficient way. Nevertheless, the above frameworks are not capable of dealing with situations in which the rejection of a specialist plays a crucial role in the decision making process. For instance, a board of five proficients is considered to choose the best aspirant in the staffing procedure and three of them rejected to deliver any decision. Although assessing the information by means of the existing tactics, the number of decision makers is vigilant to be three rather than five, i.e., the rejected proficients are totally overlooked and the decision is enclosed by means of the preferences specified by the three proficients only. It causes a significant loss of data and may lead to insufficient grades. To tackle such cases, Xu and Zhu [11] sustained a new notion named probabilistic hesitant fuzzy sets (PHFSs). The entropy is a quantitative evaluation of randomness between fuzzy sets. Termini and Luca [12] first developed the construction properties for entropy of fuzzy sets using Shannon’s probability entropy. Furthermore, some researchers [13,14,15,16] corroborated some generalized entropy measures for IFSs.

Decision making is a significant part of the real world of human beings. With the unceasing development of human beings’, real-world decision making challenges are becoming very complicated. There are often numerous aspects that have an immediate effect on the outcome of decision making problems. Such dimensions are often limited to each other and sometimes equally connected. As a consequence, the specialist must understand these things and their relationship in order to solve these decision making problems. It is a stimulating effort to make a pact with these multiattribute decision making (MADM) problems and then to make a good deal between attributes. Clearly, different attributes play an important role in choosing the best alternative between the given options, so different types of aggregation operators are used to calculate the data to characterize the DM setting. Xu [17] proposed some weighted averaging Aggregation Operators (AOs) for IFSs. Wang and Li [18] developed decision making with distance and cosine similarity measures for intuitionistic hesitant fuzzy sets (IHFSs). For instance, a complete review of the different methodologies to deal with vagueness in MADM problems is given in [19,20,21,22,23,24,25]. Meng and Chen [26] proposed correlation coefficients of hesitant fuzzy sets and their application based on fuzzy neasures. Garg [27] developed hesitant Pythagorean fuzzy Maclaurin symmetric mean operators and their applications to multiattribute decision making process. Zhao et al. [28] presented group decision making with dual hesitant fuzzy preference relations. Farhadinia and Xu [29] proposed distance and aggregation based methodologies for hesitant fuzzy decision making. Arora and Garg [30] presented a robust correlation coefficient measure of dual hesitant fuzzy soft sets and their application in decision making. In [31,32,33,34] authors proposed some other types of methodologies under the HFSs to resolve the DM problems. Yue [35] proposed an avoiding information loss approach to group decision making.

Zhang [36] developed an innovative tactic based on similarity measure for Pythagorean fuzzy multicriteria group decision making. Ren et al. [37] considered Pythagorean fuzzy TODIM tactic to multicriteria decision making. Yang et al. [38] developed Pythagorean fuzzy Choquet integral based MABAC method for multiple attribute group decision making. A hierarchical QUALIFLEX tactic is given by Zhang [39]. Pythagorean fuzzy environment measures are developed by Peng et al. [40]. Later on, generalized Pythagorean fuzzy Bonferroni mean aggregation operators are developed by Zhang et al. [41]. Liang et al. [42] generalized TOPSIS to hesitant Pythagorean fuzzy sets. PerezDomınguez et al. [43] developed MOORA for Pythagorean fuzzy information. Lately, Xue et al. [44] presented Pythagorean fuzzy LINMAP method based on the entropy for railway project investment decision making. Later on, Meng et al. [45] developed a tactic to interval-valued hesitant fuzzy multiattribute group decision making under extended Shapley-Choquet integral. Guleria et al. [46] presented Pythagorean fuzzy (R, S)-norm environment measure for multicriteria decision making problem. Moreover, Yang et al. [47] presented distance and similarity measures for hesitant fuzzy information under Hausdorff metric with applications to multicriteria decision making. Shannon [48] developed entropy in a mathematical theory of communication to determine weights of attributes. Entropy for hesitant fuzzy information under Hausdorff metric with structure of hesitant fuzzy TOPSIS was given by Yang [49].

The TOPSIS is an effective information evaluation tool, which was first given by Hwang and Yoon [50]; it is known as the approximate ideal solution. It seeks the optimal solution according to the relative closeness based on their distances from the positive ideal solution (PIS) and the negative ideal solution (NIS), so as to satisfy the nearest distance from PIS and the farthest distance from NIS. This evaluation method can effectively avoid the distortion of decision information and ensure the validity and accuracy of decision results by directly calculating the distance between PIS and NIS and ranking them accordingly. Compared with ELECTRE method, VIKOR method and other traditional methods, TOPSIS method is simple, and easy to understand and calculate, so it has been widely studied and applied by a large number of scholars. With the emergence of the form of fuzzy information, the TOPSIS method has been widely used in fuzzy environment. TOPSIS firstly prolonged the fuzzy information for resolving decision making problems by [51]. Chen [51] also presented some extensions of the TOPSIS for group decision making under a fuzzy environment.

In the recent era, different authors offered TOPSIS in different fuzzy information: like, Boran et al. [52] find out the best supplier with TOPSIS method by using the intuitionistic fuzzy information. Chen et al. [53] proposed the TOPSIS technique using interval-valued fuzzy information and also discussed the experimental analysis of the proposed technique. Li [54] presented the TOPSIS-based nonlinear-programming methodology for multiattribute decision making with interval-valued intuitionistic fuzzy sets to deal with the uncertainty in real life decision problems. Park et al. [55] introduced the TOPSIS model for decision making problems under interval-valued intuitionistic fuzzy environment. Cables et al. [56] established the TOPSIS decision making approach for linguistic variables. Xu [57] introduced the TOPSIS technique with incomplete weight information under hesitant fuzzy environment. Beg and Rashid [58] established the TOPSIS model for hesitant fuzzy linguistic term sets to deal with the uncertainty in real life complex problems. Khan et al. [59] proposed the Dombi based aggregation operators for Pythagorean fuzzy information. Biswas and Sarkar [60] established the Pythagorean fuzzy TOPSIS method with unknown weight information through entropy measure for Pythagorean fuzzy information. Barukab et al. [61] introduced the extended fuzzy TOPSIS method based on entropy measure under spherical fuzzy information.

Although there are many research results in applying the fuzzy TOPSIS method to solve MADM problems, the form of decision information used by these methods is too old and limited and cannot effectively handle current complex decision environments. Moreover, no matter what aggregation technology in fuzzy TOPSIS method we use, it may cause distortion of decision information. In addition, for decision making problems, sometimes we cannot give the weight of attributes directly and accurately, and some experts may give a too high or too low attribute evaluation value because of personal bias. In addition, the relative closeness of the traditional TOPSIS method has the defect that the nearest ideal solution to PIS is not necessarily the farthest from NIS, which makes the evaluation result inaccurate. Therefore, based on the above motivations, this paper presents an extended TOPSIS method with unknown attribute weights information under the novel idea of Pythagorean probabilistic hesitant fuzzy environment. Meanwhile, this paper proposes a weight calculation model which can effectively deal with the extreme value given by the bias expert and solve the situation of experts with large differences of opinion. Besides, using the improved relative closeness formula, the proposed method will get a more accurate evaluation result. Therefore, the innovations of this paper are mainly the following aspects: firstly, it introduces a novel concept of the Pythagorean probabilistic hesitant fuzzy set (PyPHFS). The motivation of the new concept is that in Probabilistic hesitant fuzzy set (PHFS) only positive membership degree is considered with probabilistic information, but PyPHFS is characterized by both positive hesitant membership and negative hesitant membership degrees, with the constraint that the square sum of positive and negative hesitant membership degrees is less than or equal to one. In PHFS, the DMs are limited to a particular domain and ignore the negative membership degree with its possible occurrence chances. Every negative hesitant membership degree also has some preference as compare to others. For instance, the DMs may express their opinion in DM-problems in the form of several possible values, if one DM gives values 0.3,0.4,0.6 for positive membership degree with their corresponding preference values 0.1 and 0.9, the other one may reject. The possibility of rejection level with hesitancy is considered under the proposed concept. The information of chances will decrease in spite of HFSs and PHFSs. The value of the chance of occurrence with positive and negative membership degrees gives more details on the level of difference of opinion of the DMs. To deal with the uncertainty in decision making problems, the novel concept of Pythagorean probabilistic hesitant fuzzy set is proposed. The originality of the paper is given as follows:

(1) The PHFS is generalized by PyPHFS.
(2) The TOPSIS method is generalized by PyPHFS with unidentified weight information.
(3) The PyPHF-TOPSIS method is proposed to solve the PyPHF-MCDM problems with unknown weight information.
(4) We extend the distance measures and aggregation operators and apply it to the MCDM problem.
(5) A case study for the most crucial fog-haze influence factor is provided to show the effectiveness and applicability of the proposed approach.

The arrangement of the paper is as follows. Section 2 gives a review of FSs, IFSs, PyFSs, HFSs and PyHFSs. Section 3 gives some discussion about the algebraic operations of PyPHFSs. In Section 4 we exhibit distance measure and weighted distance measure, together with its properties. In Section 5, the novel technique to handle vagueness in DM problems to sort out the finest alternative according to a list of attributes is proposed. Section 6 explains the application of the proposed MCDM method. In Section 7, a conclusion and discussion of the manuscript is given.

## 2. Preliminaries

Let us remember the fundamentals of fuzzy sets, intuitionistic fuzzy sets and Pythagorean fuzzy sets in this section for a moment. Upon review, these notions will be used here.

**Definition** **1** ([3]). *For a fixed set $\overset{˘}{E}$. A FS ε in $\overset{˘}{E}$ is presented as*

$$\varepsilon =\left\{\langle \ell_{x},\rho_{\varepsilon }\left(\ell_{x}\right)\rangle |\ell_{x}\in \overset{˘}{E}\right\},$$

*for each $\ell_{x}\in \overset{˘}{E},$ the positive membership grade $\rho_{\varepsilon }:\overset{˘}{E}\to \Theta$ specifies the degree to which the element $\ell_{x}$ belongs to the fuzzy set ε, where $\Theta =\left[0,1\right]$ be the unit interval.*

**Definition** **2** ([4]). *For a fixed set $\overset{˘}{E}$. An IFS ε in $\overset{˘}{E}$ is presented as*

$$\varepsilon =\left\{\langle \ell_{x},\rho_{\varepsilon }\left(\ell_{x}\right),{\tilde{n}}_{\varepsilon }\left(\ell_{x}\right)\rangle |\ell_{x}\in \overset{˘}{E}\right\},$$

*for each $\ell_{x}\in \overset{˘}{E}$, the positive membership grade $\rho_{\varepsilon }:\overset{˘}{E}\to \Theta$ and the negative membership grade ${\tilde{n}}_{\varepsilon }:\overset{˘}{E}\to \Theta$ specifies the degree of positive and negative membership of the element $\ell_{x}$ to the Intuitionistic fuzzy set ε, respectively, where $\Theta =\left[0,1\right]$ is the unit interval. Moreover, it is required that $0\leq \rho_{\varepsilon }\left(\ell_{x}\right)+{\tilde{n}}_{\varepsilon }\left(\ell_{x}\right)\leq 1,$ for each $\ell_{x}\in \overset{˘}{E}$.*

**Definition** **3** ([5]). *For a fixed set $\overset{˘}{E}$. A PyFS ε in $\overset{˘}{E}$ is presented as*

$$\varepsilon =\left\{\langle \ell_{x},\rho_{\varepsilon }\left(\ell_{x}\right),{\tilde{n}}_{\varepsilon }\left(\ell_{x}\right)\rangle |\ell_{x}\in \overset{˘}{E}\right\},$$

*for each $\ell_{x}\in \overset{˘}{E},$ the positive membership grade $\rho_{\varepsilon }:\overset{˘}{E}\to \Theta$ and the negative membership grade ${\tilde{n}}_{\varepsilon }:\overset{˘}{E}\to \Theta$ specifies the degree of positive and negative membership of the element $\ell_{x}$ to the Pythagorean fuzzy set ε, respectively, where $\Theta =\left[0,1\right]$ is the unit interval. Furthermore, it is required that $0\leq \rho_{\varepsilon }^{2}\left(\ell_{x}\right)+{\tilde{n}}_{\varepsilon }^{2}\left(\ell_{x}\right)\leq 1$, for each $\ell_{x}\in \overset{˘}{E}$.*

*Conventionally, $\chi_{\varkappa }=\sqrt{1-\rho_{\varepsilon }^{2}\left(\ell_{x}\right)-{\tilde{n}}_{\varepsilon }^{2}\left(\ell_{x}\right)}$ is said to be degree of hesitancy of $\ell_{x}$ to ε. In what follows, we symbolize by $PyF\hat{S}\left(\overset{˘}{E}\right)$ the collection of all Pythagorean fuzzy sets in $\overset{˘}{E}$. For ease, we shall symbolize the Pythagorean fuzzy number (PyFN) by the pair $\varepsilon =\left(\rho_{\varepsilon },{\tilde{n}}_{\varepsilon }\right).$*

**Definition** **4** ([5]). *Let $\varepsilon_{1},\varepsilon_{2}\in PyF\hat{S}\left(\overset{˘}{E}\right)$. Then*

(1) $\varepsilon_{1}⊑\varepsilon_{2}$ if and only if $\rho_{\varepsilon_{1}}\left(\ell_{x}\right)\leq \rho_{\varepsilon_{2}}\left(\ell_{x}\right)$ and ${\tilde{n}}_{\varepsilon_{1}}\left(\ell_{x}\right)\geq {\tilde{n}}_{\varepsilon_{2}}\left(\ell_{x}\right)$ for each $\ell_{x}\in \overset{˘}{E}.$ Clearly $\varepsilon_{1}=\varepsilon_{2}$ if $\varepsilon_{1}⊑\varepsilon_{2}$ and $\varepsilon_{2}⊑\varepsilon_{1}.$
(2) $\varepsilon_{1}⊓\varepsilon_{2}=\left\{\min\left(\rho_{\varepsilon_{1}}\left(\ell_{x}\right),\rho_{\varepsilon_{2}}\left(\ell_{x}\right)\right),\max\left({\tilde{n}}_{\varepsilon_{1}}\left(\ell_{x}\right),{\tilde{n}}_{\varepsilon_{2}}\left(\ell_{x}\right)\right)|\ell_{x}\in \overset{˘}{E}\right\},$
(3) $\varepsilon_{1}⊔\varepsilon_{2}=\left\{\max\left(\rho_{\varepsilon_{1}}\left(\ell_{x}\right),\rho_{\varepsilon_{2}}\left(\ell_{x}\right)\right),\min\left({\tilde{n}}_{\varepsilon_{1}}\left(\ell_{x}\right),{\tilde{n}}_{\varepsilon_{2}}\left(\ell_{x}\right)\right)|\ell_{x}\in \overset{˘}{E}\right\},$
(4) $\varepsilon_{1}^{c}=\left\{{\tilde{n}}_{\varepsilon_{1}}\left(\ell_{x}\right),\rho_{\varepsilon_{1}}\left(\ell_{x}\right)|\ell_{x}\in \overset{˘}{E}\right\}.$

**Definition** **5** ([9]). *For a fixed set $\overset{˘}{E}$. A Hesitant Fuzzy Set (HFS) ε in $\overset{˘}{E}$ is presented as*

$$\varepsilon =\left\{\langle \ell_{x},h_{\varkappa }\left(\ell_{x}\right)\rangle |\ell_{x}\in \overset{˘}{E}\right\}$$

*where $h_{\varkappa }\left(\ell_{x}\right)$ is in the form of set which contained some possible values in unit interval, i.e.,$\left[0,1\right]$ which represent the membership degree of $\ell_{x}\in \overset{˘}{E}$ in ε.*

**Definition** **6** ([9]). *Let $\varepsilon_{1},\varepsilon_{2}\in HFS\left(\overset{˘}{E}\right).$ Then*

*(1)*  $\varepsilon_{1}^{c}={⨆}_{\alpha \in h_{\varepsilon_{1}}\left(\ell_{x}\right)}\left\{1-\alpha \right\};$
*(2)*  $\varepsilon_{1}⊔\varepsilon_{2}=h_{\varepsilon_{1}}\left(\ell_{x}\right)⊻h_{\varepsilon_{2}}\left(\ell_{x}\right)={⨆}_{\alpha_{1}\in h_{\varepsilon_{1}}\left(\ell_{x}\right),\alpha_{2}\in h_{\varepsilon_{2}}\left(\ell_{x}\right)}\max\left\{\alpha_{1},\alpha_{2}\right\};$
*(3)*  $\varepsilon_{1}⊓\varepsilon_{2}=h_{\varepsilon_{1}}\left(\ell_{x}\right)⊼h_{\varepsilon_{2}}\left(\ell_{x}\right)={⨆}_{\alpha_{1}\in h_{\varepsilon_{1}}\left(\ell_{x}\right),\alpha_{2}\in h_{\varepsilon_{2}}\left(\ell_{x}\right)}\min\left\{\alpha_{1},\alpha_{2}\right\};$

**Definition** **7** ([10]). *For a fixed set $\overset{˘}{E}$. A PyHFS ε in $\overset{˘}{E}$ is presented as*

$$\varepsilon =\left\{\langle \ell_{x},\rho_{h_{\varkappa }}\left(\ell_{x}\right),{\tilde{n}}_{h_{\varkappa }}\left(\ell_{x}\right)\rangle |\ell_{x}\in \overset{˘}{E}\right\},$$

*for each $\ell_{x}\in \overset{˘}{E},$ the positive membership grade $\rho_{\varepsilon }$ and the negative membership grade ${\tilde{n}}_{\varkappa }$ are sets in some values in $\left[0,1\right],$ specifies the possible degree of positive and negative membership of the element $\ell_{x}$ to the Pythagorean hesitant fuzzy set ε, respectively. Furthermore, it is required that ${\left(\max\left(\rho_{h_{\varkappa }}\left(\ell_{x}\right)\right)\right)}^{2}+{\left(\min\left({\tilde{n}}_{h_{\varkappa }}\left(\ell_{x}\right)\right)\right)}^{2}\leq 1$ and ${\left(\min\left(\rho_{h_{\varkappa }}\left(\ell_{x}\right)\right)\right)}^{2}+{\left(\max\left({\tilde{n}}_{h_{\varkappa }}\left(\ell_{x}\right)\right)\right)}^{2}\leq 1.$ For ease, we shall symbolize the Pythagorean Hesitant Fuzzy Number (PyHFN) by the pair $\varepsilon =\left(\rho_{h_{\varkappa }},{\tilde{n}}_{h_{\varkappa }}\right).$*

**Definition** **8** ([11]). *For a fixed set $\overset{˘}{E}$. A Probabilistic Hesitant Fuzzy Set (PHFS) ε in $\overset{˘}{E}$ is presented as*

$$\varepsilon =\left\{\langle \ell_{x},h_{\varkappa }\left(\ell_{x}\right)/p_{x}\rangle |\ell_{x}\in \overset{˘}{E}\right\}$$

*where $h_{\varkappa }\left(\ell_{x}\right)$ is subset of $\left[0,1\right]$ and $h_{\varkappa }\left(\ell_{x}\right)/p_{x}$ represent the membership degree of $\ell_{x}\in \overset{˘}{E}$ in ε. Furthermore, $p_{x}$ represent the possibilities of $\;h_{\varkappa }\left(\ell_{x}\right)$, with condition that $\sum_{x}p_{x}=1.$*

## 3. Pythagorean Probabilistic Hesitant Fuzzy Sets and Their Operational Laws

In this segment, we develop the novel hybrid notion of Pythagorean Probabilistic Hesitant Fuzzy Sets (PyPHFSs) to deal with the uncertainty in decision support systems. We also introduce the basic operational laws for PyPHFSs.

**Definition** **9.** *For a fixed set $\overset{˘}{E}$. A PyPHFS ε in $\overset{˘}{E}$ is presented as*

$$\varepsilon =\left\{\langle \ell_{x},\rho_{h_{\varkappa }}\left(\ell_{x}\right)/p_{x},{\tilde{n}}_{h_{\varkappa }}\left(\ell_{x}\right)/q_{x}\rangle |\ell_{x}\in \overset{˘}{E}\right\},$$

*for each $\ell_{x}\in \overset{˘}{E},$$\rho_{h_{\varkappa }}\left(\ell_{x}\right)$ and ${\tilde{n}}_{h_{\varkappa }}\left(\ell_{x}\right)$ are sets of some values in $\left[0,1\right]$. Where $\rho_{h_{\varkappa }}\left(\ell_{x}\right)/p_{x}$ & ${\tilde{n}}_{h_{\varkappa }}\left(\ell_{x}\right)/q_{x}$ specifies the possible degree of positive and negative membership of the element $\ell_{x}$ to the Pythagorean probabilistic hesitant fuzzy set ε, respectively. $p_{x}$ and $q_{x}$ represent the possibilities of membership grades. In addition, there is 0≤$\gamma_{i},,\eta_{j}\leq 1$ and $0\leq p_{i},q_{j}\leq 1$ with $\sum_{i=1}^{L}p_{i}\leq 1,\sum_{j=1}^{L}q_{j}\leq 1$(L is a positive integer to describe the number of elements contained in PyPHFS), where $\gamma_{i}\in \rho_{h_{\varkappa }}\left(\ell_{x}\right),\eta_{j}\in {\tilde{n}}_{h_{\varkappa }}\left(\ell_{x}\right),p_{i}\in p_{x},q_{j}\in q_{x}$. Furthermore, it is required that ${\left(\max\left(\rho_{h_{\varkappa }}\left(\ell_{x}\right)\right)\right)}^{2}+{\left(\min\left({\tilde{n}}_{h_{\varkappa }}\left(\ell_{x}\right)\right)\right)}^{2}\leq 1$ and ${\left(\min\left(\rho_{h_{\varkappa }}\left(\ell_{x}\right)\right)\right)}^{2}+{\left(\max\left({\tilde{n}}_{h_{\varkappa }}\left(\ell_{x}\right)\right)\right)}^{2}\leq 1.$ For ease, we shall symbolize the Pythagorean Probabilistic Hesitant Fuzzy Number (PyPHFN) by the pair $\varepsilon =\left(\rho_{h_{\varkappa }}/p_{x},{\tilde{n}}_{h_{\varkappa }}/q_{x}\right).$*

The collection of all Pythagorean probabilistic hesitant fuzzy sets in $\overset{˘}{E}$ is symbolized by $PyPHF\hat{S}\left(\overset{˘}{E}\right)$

**Example** **1.** *For a fixed set $\overset{˘}{E}=\left\{\ell_{1},\ell_{2}\right\}$. A $\varepsilon \in PyPHF\hat{S}\left(\overset{˘}{E}\right)$, i.e.,*

$$\varepsilon =\left\{\begin{array}{c} \left(\ell_{1},\left(0.2/0.3,0.5/0.2,0.9/0.5\right),\left(0.1/0.4,0.3/0.5\right)\right),\\ \left(\ell_{2},\left(0.4/0.6,0.7/0.4\right),\left(0.6/0/4,0.2/0.4,0.3/0.2\right)\right) \end{array}\right\},$$

*where $0\leq p_{x}\leq 1$ with $\sum_{x=1}^{L}p_{x}\leq 1.$ Then*

$$\begin{array}{cc} \max\rho_{h_{1}}\left(\ell_{1}\right)=\max\left\{0.2,0.5,0.9\right\} & \max{\tilde{n}}_{h_{1}}\left(\ell_{1}\right)=\max\left\{0.1,0.3\right\}\\ =0.9 & =0.3\\ \max\rho_{h_{2}}\left(\ell_{2}\right)=\max\left\{0.4,0.7\right\} & \max{\tilde{n}}_{h_{2}}\left(\ell_{2}\right)=\max\left\{0.6,0.2,0.3\right\}\\ =0.7 & =0.6\\ \min\rho_{h_{1}}\left(\ell_{1}\right)=\min\left\{0.2,0.5,0.9\right\} & \min{\tilde{n}}_{h_{1}}\left(\ell_{1}\right)=\min\left\{0.1,0.3\right\}\\ =0.2 & =0.1\\ \min\rho_{h_{2}}\left(\ell_{2}\right)=\min\left\{0.4,0.7\right\} & \min{\tilde{n}}_{h_{2}}\left(\ell_{2}\right)=\min\left\{0.6,0.2,0.3\right\}\\ =0.4 & =0.2 \end{array}$$

*it is clear that*

$$\begin{array}{ccc} {\left(\max\left(\rho_{h_{1}}\left(\ell_{1}\right)\right)\right)}^{2}+{\left(\min\left({\tilde{n}}_{h_{1}}\left(\ell_{1}\right)\right)\right)}^{2} & = & {\left(0.9\right)}^{2}+{\left(0.1\right)}^{2}\\ & = & 0.82\leq 1 \end{array}$$

*and*

$$\begin{array}{ccc} {\left(\min\left(\rho_{h_{1}}\left(\ell_{1}\right)\right)\right)}^{2}+{\left(\max\left({\tilde{n}}_{h_{1}}\left(\ell_{1}\right)\right)\right)}^{2} & = & {\left(0.2\right)}^{2}+{\left(0.3\right)}^{2}\\ & = & 0.13\leq 1 \end{array}$$

*So, $\left(\rho_{h_{1}}\left(\ell_{1}\right)/p,{\tilde{n}}_{h_{1}}\left(\ell_{1}\right)/q\right)$ is a PyPHFN. Similarly, $\left(\rho_{h_{2}}\left(\ell_{2}\right)/p,{\tilde{n}}_{h_{2}}\left(\ell_{2}\right)/q\right)$ is a PyPHFN. Thus, $\varepsilon \in PyPHϝ\hat{S}\overset{˘}{E}.$*

**Definition** **10.** *Let $\varepsilon_{1}=\left(\rho_{h_{x_{1}}}/p_{x_{{}_{1}}},{\tilde{n}}_{h_{x_{1}}}/q_{x_{{}_{1}}}\right)$ and $\varepsilon_{2}=\left(\rho_{h_{x_{2}}}/p_{x_{2}},{\tilde{n}}_{h_{{}_{x_{2}}}}/q_{x_{{}_{2}}}\right)$ be two PyPHFNs. The basic operational laws defined as*

*(1)*  $\varepsilon_{1}\cup \varepsilon_{2}=\left\{{⨆}_{\gamma_{1}\in \rho_{h_{{}_{x_{1}}}\left(l_{x}\right)},p_{1\in p_{x_{1}}}}\left(\max\left(\gamma_{1}/p_{1},\gamma_{2}/p_{2}\right)\right),{⨆}_{\eta_{{}_{1}}\in {\tilde{n}}_{{}_{h_{x_{1}}}\left(l_{x}\right),q_{1}\in }q_{x_{2}}}\left(\min\left(\eta_{1}/q_{1},\eta_{2}/q_{2}\right)\right)\right\};$
*(2)*  $\varepsilon_{1}\cap \varepsilon_{2}=\left\{{⨆}_{\gamma_{1}\in \rho_{h_{{}_{x_{1}}}\left(l_{x}\right)},p_{1\in p_{x_{1}}}}\left(\min\left(\gamma_{1}/p_{1},\gamma_{2}/p_{2}\right)\right),{⨆}_{\eta_{{}_{1}}\in {\tilde{n}}_{{}_{h_{x_{1}}}\left(l_{x}\right),q_{1}\in }q_{x_{2}}}\left(\max\left(\eta_{1}/q_{1},\eta_{2}/q_{2}\right)\right)\right\};$
*(3)*  $\varepsilon_{1}^{c}=\left\{{\tilde{n}}_{h_{\varkappa }}/q_{x},\rho_{h_{\varkappa }}/p_{x}\right\}$

**Definition** **11.** *Let $\varepsilon_{1}=\left(\rho_{h_{x_{1}}}/p_{x_{{}_{1}}},{\tilde{n}}_{h_{x_{1}}}/q_{x_{{}_{1}}}\right)$ and $\varepsilon_{2}=\left(\rho_{h_{x_{2}}}/p_{x_{2}},{\tilde{n}}_{h_{{}_{x_{2}}}}/q_{x_{{}_{2}}}\right)$ be two PyPHFNs and ζ>0(∈R), then their operations are presented as:*

*(1)*  $\varepsilon_{1}⊕\varepsilon_{2}=\left\{\begin{array}{c} {⨆}_{{}_{\gamma_{1}\in \rho_{h_{{}_{x_{1}}}\left(l_{x}\right)},\gamma_{2}\in \rho_{{}_{h_{x_{2}}}\left(l_{x}\right),p_{1}\in p_{x_{{}_{1}}},p_{2}\in }p_{x_{2}}}}\left(\sqrt{\gamma_{1}^{2}+\gamma_{2}^{2}-\gamma_{1}^{2}\gamma_{2}^{2}}/p_{1}p_{2}\right),\\ {⨆}_{\eta_{1}\in {\tilde{n}}_{h_{{}_{x_{1}}}\left(l_{x}\right)},\eta_{{}_{2}}\in {\tilde{n}}_{{}_{h_{x_{2}}}\left(l_{x}\right),q_{1}\in q_{x_{{}_{1}}},q_{2}\in }q_{x_{2}}}\left(\eta_{1}\eta_{2}/q_{1}q_{2}\right) \end{array}\right\};$
*(2)*  $\varepsilon_{1}⊗\varepsilon_{2}=\left\{\begin{array}{c} {⨆}_{{}_{\gamma_{1}\in \rho_{h_{{}_{x_{1}}}\left(l_{x}\right)},\gamma_{2}\in \rho_{{}_{h_{x_{2}}}\left(l_{x}\right),p_{1}\in p_{x_{{}_{1}}},p_{2}\in }p_{x_{2}}}}\left(\gamma_{1}\gamma_{2}/p_{1}p_{2}\right),\\ {⨆}_{\eta_{1}\in {\tilde{n}}_{h_{{}_{x_{1}}}},\eta_{{}_{2}}\in {\tilde{n}}_{{}_{h_{x_{2}}},q_{1}\in q_{x_{{}_{1}}},q_{2}\in }q_{x_{2}}}\left(\sqrt{\eta_{1}^{2}+\eta_{2}^{2}-\eta_{1}^{2}\eta_{2}^{2}}/q_{1}q_{2}\right) \end{array}\right\};$
*(3)*  $\zeta \varepsilon_{1}=\left\{{⨆}_{\gamma_{1}\in \rho_{h_{{}_{x_{1}}}\left(l_{x}\right)},p_{1\in p_{x_{1}}}}\left(\sqrt{1-{\left(1-\gamma_{1}^{2}\right)}^{\zeta }}/p_{1}\right),{⨆}_{{}_{\eta_{{}_{1}}\in {\tilde{n}}_{{}_{h_{x_{1}}}\left(l_{x}\right),q_{1}\in }q_{x_{2}}}}\left(\eta_{1}^{\zeta }/q_{1}\right)\right\};$
*(4)*  $\varepsilon_{1}^{\zeta }=\left\{{⨆}_{\gamma_{1}\in \rho_{h_{{}_{x_{1}}}\left(l_{x}\right)},p_{1\in p_{x_{1}}}}\left(\gamma_{1}^{\zeta }/p_{1}\right),{⨆}_{{}_{\eta_{{}_{1}}\in {\tilde{n}}_{{}_{h_{x_{1}}}\left(l_{x}\right),q_{1}\in }q_{x_{2}}}}\left(\sqrt{1-{\left(1-\eta_{1}^{2}\right)}^{\zeta }}/q_{1}\right)\right\}.$

**Definition** **12.** *For any PyPHFN $\varepsilon =\left(\rho_{h_{\varkappa }}/p_{x},{\tilde{n}}_{h_{\varkappa }}/q_{x}\right)$, a score function is defined as*

$$s\left(\varepsilon \right)={\left(\frac{1}{M_{\varepsilon }}\sum_{\gamma_{i}\in \rho_{h_{x},p_{i}\in p_{h_{x}}}}\left(\gamma_{i}\cdot p_{i}\right)\right)}^{2}-{\left(\frac{1}{N_{\varepsilon }}\sum_{\eta_{i}\in {\tilde{n}}_{h_{\varkappa }},q_{i}\in q_{h_{x}}}\left(\eta_{i}\cdot q_{i}\right)\right)}^{2}$$

*where $M_{\varepsilon }$ denotes the number of elements in $\rho_{h_{\varkappa }}$ and $N_{\varepsilon }$ denotes the number of elements in ${\tilde{n}}_{h_{\varkappa }}.$*

**Definition** **13.** *For any PyPHFN $\varepsilon =\left(\rho_{h_{\varkappa }}/p_{x},{\tilde{n}}_{h_{\varkappa }}/q_{x}\right)$, an accuracy function is defined as*

$$h\left(\varepsilon \right)={\left(\frac{1}{M_{\varepsilon }}\sum_{\gamma_{i}\in \rho_{h_{x},p_{i}\in p_{h_{x}}}}\left(\gamma_{i}\cdot p_{i}\right)\right)}^{2}+{\left(\frac{1}{N_{\varepsilon }}\sum_{\eta_{i}\in {\tilde{n}}_{h_{\varkappa }},q_{i}\in q_{h_{x}}}\left(\eta_{i}\cdot q_{i}\right)\right)}^{2}$$

*where $M_{\varepsilon }$ denotes the number of elements in $\rho_{h_{\varkappa }}$ and $N_{\varepsilon }$ denotes the number of elements in ${\tilde{n}}_{h_{\varkappa }}.$*

**Definition** **14.** *Let $\varepsilon_{1}=\left(\rho_{h_{x_{1}}}/p_{x_{1}},{\tilde{n}}_{h_{x_{1}}}/q_{x_{1}}\right)$ and $\varepsilon_{2}=\left(\rho_{h_{x_{2}}}/p_{x_{2}},{\tilde{n}}_{h_{x_{2}}}/q_{x_{2}}\right)$ be two PyPHFNs. Then by using the above definitions, comparison of PyPHFNs can be described as*

*(1)*  *If $s\left(\varepsilon_{1}\right)>s\left(\varepsilon_{2}\right)$, then $\varepsilon_{1}>\varepsilon_{2}.$*
*(2)*  *If $s\left(\varepsilon_{1}\right)=s\left(\varepsilon_{2}\right)$, and $h\left(\varepsilon_{1}\right)>h\left(\varepsilon_{2}\right)$ then $\varepsilon_{1}>\varepsilon_{2}$*

## 4. Methodological Development of Pythagorean Probabilistic Hesitant Fuzzy Entropy Measure

In this segment, the proposed extended distance measures and the weighted extended distance measures for PyPHFSs are defined. Then, a proposed entropy measure for PyPHFS is presented for quantitative evaluation of randomness of a PyPHFS.

### 4.1. Distance Measure for Pyphfss

**Definition** **15.** *For any two PyPHFSs $\varepsilon =\left\{\rho_{\varepsilon_{i}}/p_{\varepsilon_{i}},{\tilde{n}}_{\varepsilon_{j}}/q_{\varepsilon_{j}}\right\}$ and $\vartheta =\{\rho_{\vartheta_{\tilde{ı}}}/p_{\vartheta_{\tilde{ı}}},{\tilde{n}}_{\vartheta_{\hat{j}}}/q_{\vartheta_{\hat{j}}}\}$, where $i=1,2,\ldots ,M_{\varepsilon };$$j=1,2,\ldots ,N_{\varepsilon };$$\tilde{ı}=1,2,\ldots ,M_{\vartheta }$ and $\hat{j}=1,2,\ldots ,N_{\vartheta }.$ For a real number ζ>0, we defined distance measure between ε and ϑ as:*

$$d^{\circ }\left(\varepsilon ,\vartheta \right)={\left(\sum_{x=1}^{n}\frac{1}{2n}\left(\begin{array}{c} {\left|\frac{1}{M_{\varepsilon }}\sum_{i=1}^{M_{\varepsilon }}{\left(\gamma_{\varepsilon_{i}}p_{\varepsilon_{i}}\right)}^{2}-\frac{1}{M_{\vartheta }}\sum_{\tilde{ı}=1}^{M_{\vartheta }}{\left(\gamma_{\vartheta_{\tilde{ı}}}p_{\vartheta_{\tilde{ı}}}\right)}^{2}\right|}^{\zeta }+\\ {\left|\frac{1}{N_{\varepsilon }}\sum_{j=1}^{N_{\varepsilon }}{\left(\eta_{\varepsilon_{j}}q_{\varepsilon_{j}}\right)}^{2}-\frac{1}{N_{\vartheta }}\sum_{\hat{j}=1}^{N_{\vartheta }}{\left(\eta_{\vartheta_{\hat{j}}}q_{\vartheta_{\hat{j}}}\right)}^{2}\right|}^{\zeta } \end{array}\right)\right)}^{\frac{1}{\zeta }},$$

*where $\gamma_{\varepsilon_{i}}\in \rho_{\varepsilon_{i}},\gamma_{\vartheta_{\tilde{ı}}}\in \rho_{\vartheta_{\tilde{ı}}},\eta_{\varepsilon_{j}}\in {\tilde{n}}_{\varepsilon_{j}},\eta_{\vartheta_{\hat{j}}}\in {\tilde{n}}_{\vartheta_{\hat{j}}}.$*

**Definition** **16.** *For any two PyPHFSs $\varepsilon =\left\{\rho_{\varepsilon_{i}}/p_{\varepsilon_{i}},{\tilde{n}}_{\varepsilon_{j}}/q_{\varepsilon_{j}}\right\}$ and $\vartheta =\{\rho_{\vartheta_{\tilde{ı}}}/p_{\vartheta_{\tilde{ı}}},{\tilde{n}}_{\vartheta_{\hat{j}}}/q_{\vartheta_{\hat{j}}}\}$, where $i=1,2,\ldots ,M_{\varepsilon };$$j=1,2,\ldots ,N_{\varepsilon };$$\tilde{ı}=1,2,\ldots ,M_{\vartheta }$ and $\hat{j}=1,2,\ldots ,N_{\vartheta }.$ For a real number ζ>0, we defined weighted distance measure between ε and ϑ as:*

$$d^{\circ }\left(\varepsilon ,\vartheta \right)={\left(\sum_{x=1}^{n}\frac{1}{2n}\omega_{x}\left(\begin{array}{c} {\left|\frac{1}{M_{\varepsilon }}\sum_{i=1}^{M_{\varepsilon }}{\left(\gamma_{\varepsilon_{i}}p_{\varepsilon_{i}}\right)}^{2}-\frac{1}{M_{\vartheta }}\sum_{\tilde{ı}=1}^{M_{\vartheta }}{\left(\gamma_{\vartheta_{\tilde{ı}}}p_{\vartheta_{\tilde{ı}}}\right)}^{2}\right|}^{\zeta }+\\ {\left|\frac{1}{N_{\varepsilon }}\sum_{j=1}^{N_{\varepsilon }}{\left(\eta_{\varepsilon_{j}}q_{\varepsilon_{j}}\right)}^{2}-\frac{1}{N_{\vartheta }}\sum_{\hat{j}=1}^{N_{\vartheta }}{\left(\eta_{\vartheta_{\hat{j}}}q_{\vartheta_{\hat{j}}}\right)}^{2}\right|}^{\zeta } \end{array}\right)\right)}^{\frac{1}{\zeta }},$$

*where $\omega_{x}={\left(\omega_{1},\ldots ,\omega_{n}\right)}^{T}$ is the weight vector and $\gamma_{\varepsilon_{i}}\in \rho_{\varepsilon_{i}},\gamma_{\vartheta_{\tilde{ı}}}\in \rho_{\vartheta_{\tilde{ı}}},\eta_{\varepsilon_{j}}\in {\tilde{n}}_{\varepsilon_{j}},\eta_{\vartheta_{\hat{j}}}\in {\tilde{n}}_{\vartheta_{\hat{j}}}.$*

**Theorem** **1.** *Let ε and ϑ be two PyPHFSs, then the distance measure has the following constraints:*

*(P1) $0\leq d^{\circ }\left(\varepsilon ,\vartheta \right)\leq 1;$*
*(P2) $d^{\circ }\left(\varepsilon ,\vartheta \right)=d^{\circ }\left(\vartheta ,\varepsilon \right);$*
*(P3) $d^{\circ }\left(\varepsilon ,\vartheta \right)=0⟺\varepsilon =\vartheta ;$*

**Proof.**  Straight forward □

### 4.2. Entropy Measure for Pyphfss

The weight information of attributes/criteria is very important in decision making problems. Many scholars focus on decision making problems with incomplete or unknown attributes weight information in different fuzzy environments [62,63]. Entropy [48] is a conventional term from information theory which is also used to determine weight of attributes. The larger the value of entropy in a given attribute is, the smaller the differences in the ratings of alternatives with respect to this attribute. In turn, this means that this kind of attribute supplies less information and has a smaller weight.

**Definition** **17.** *The information of Shannon entropy $E\left(E_{1},E_{2},E_{3},\ldots ,E_{m}\right)$ for PyPHFS is defined as*

$$\frac{-1}{\ln\left(m\right)}\sum_{i=1}^{m}\left(\rho_{h_{\varkappa }}\left(\ln\rho_{h_{\varkappa }}\right)\times p_{x}+{\tilde{n}}_{h_{\varkappa }}\left(\ln{\tilde{n}}_{h_{\varkappa }}\right)\times q_{x}\right)$$

#### Properties of Entropy Measure

For any two PyPHFSs ε and ϑ, the entropy measure $E\left(\varepsilon \right)$ and $E\left(\vartheta \right)$ fulfill the given axioms:

(a) $E\left(\varepsilon \right)=0⟺\varepsilon$ is a crisp set.
(b) $E\left(\varepsilon \right)=1⟺\gamma_{\varepsilon_{j}}\left(\ell_{x}\right)=\eta_{\varepsilon_{j}}\left(\ell_{x}\right)\forall \ell_{x}\in \overset{˘}{E}$
(c) $E\left(\varepsilon \right)\leq E\left(\vartheta \right)$ if $\varepsilon {⪯}_{PyPHFS}\vartheta$
(d) $E\left(\varepsilon \right)=E\left(\varepsilon^{c}\right)\;$

## 5. Pythagorean Probabilistic Hesitant Fuzzy Mcdm Topsis Technique

Let $\bar{A}=\left\{{\bar{A}}_{1},{\bar{A}}_{2},\ldots ,{\bar{A}}_{k}\right\}$ be a set of alternatives, $\hat{C}=\left\{{\hat{C}}_{1},{\hat{C}}_{2},\ldots ,{\hat{C}}_{n}\right\}$ be a set of criteria and *r* be the number of DMs, DM_*e*_ $\left(e=1,2,\ldots ,r\right)$, to show their perception about k alternatives with respect to the *n* criteria by taking $\varepsilon_{ij}^{\left(e\right)}=\left(\rho_{ij}^{\left(e\right)},{\tilde{n}}_{ij}^{\left(e\right)}\right)$. The decision matrix of the eth decision maker is presented as:

$$\varepsilon^{\left(e\right)}={\left[\varepsilon_{ij}^{\left(e\right)}\right]}_{k\times n}={\left[\left(\rho_{\varepsilon_{ij}}^{\left(e\right)}/p_{\varepsilon_{ij}},{\tilde{n}}_{\varepsilon_{ij}}^{\left(e\right)}/q_{\varepsilon_{ij}}\right)\right]}_{k\times n}$$

where

$$\varepsilon^{\left(e\right)}=\begin{array}{c} {\bar{A}}_{1}\\ {\bar{A}}_{2}\\ \ldots \\ {\bar{A}}_{k} \end{array}\overset{{\hat{C}}_{1}\;\;\;\;\;\;\;{\hat{C}}_{2}\;\;\;\;\;\;\;\;\;\;\;\;\;\ldots \;\;\;\;\;\;\;{\hat{C}}_{n}}{\left[\begin{array}{cccc} \left(\rho_{\varepsilon_{11}}^{\left(e\right)}/p_{\varepsilon_{11}},{\tilde{n}}_{\varepsilon_{11}}^{\left(e\right)}/q_{\varepsilon_{11}}\right)\; & \left(\rho_{\varepsilon_{12}}^{\left(e\right)}/p_{\varepsilon_{12}},{\tilde{n}}_{\varepsilon_{12}}^{\left(e\right)}/q_{\varepsilon_{12}}\right)\; & \ldots & \left(\rho_{\varepsilon_{1n}}^{\left(e\right)}/p_{\varepsilon_{1n}},{\tilde{n}}_{\varepsilon_{1n}}^{\left(e\right)}/q_{\varepsilon_{1n}}\right)\\ \left(\rho_{\varepsilon_{21}}^{\left(e\right)}/p_{\varepsilon_{21}},{\tilde{n}}_{\varepsilon_{21}}^{\left(e\right)}/q_{\varepsilon_{21}}\right)\; & \left(\rho_{\varepsilon_{22}}^{\left(e\right)}/p_{\varepsilon_{22}},{\tilde{n}}_{\varepsilon_{22}}^{\left(e\right)}/q_{\varepsilon_{22}}\right)\; & \ldots & \left(\rho_{\varepsilon_{2n}}^{\left(e\right)}/p_{\varepsilon_{2n}},{\tilde{n}}_{\varepsilon_{2n}}^{\left(e\right)}/q_{\varepsilon_{2n}}\right)\\ \ldots & \ldots & \ldots & \ldots \\ \left(\rho_{\varepsilon_{k1}}^{\left(e\right)}/p_{\varepsilon_{k1}},{\tilde{n}}_{\varepsilon_{k1}}^{\left(e\right)}/q_{\varepsilon_{k1}}\right)\; & \left(\rho_{\varepsilon_{k2}}^{\left(e\right)}/p_{\varepsilon_{k2}},{\tilde{n}}_{\varepsilon_{k2}}^{\left(e\right)}/q_{\varepsilon_{k2}}\right)\; & \ldots & \left(\rho_{\varepsilon_{kn}}^{\left(e\right)}/p_{\varepsilon_{kn}},{\tilde{n}}_{\varepsilon_{kn}}^{\left(e\right)}/q_{\varepsilon_{kn}}\right) \end{array}\right]}$$

### Pyphf-Topsis Method

This method has two main parts. Firstly, a method based on TOPSIS for calculating the weights of criteria/attributes utilizing the proposed entropy measure for PyPHFNs is presented. In the last part, we consider a ranking procedure based on degree of similarity to ideal solution. Steps for solving the PyPHF MCDM problem are presented as follows:

Step 1. Firstly, we collect the information in the form of PyPHFNs given by the decision makers (DMs).
Step 2. In this step, we normalized the data specified by DMs, since the decision matrix may have some benefit and cost criteria altogether, as follows:
  $$\begin{array}{ccc} g_{ij}^{\left(e\right)} & = & \left\{\begin{array}{cc} \varepsilon_{ij}^{\left(e\right)} & \mathrm{for}\;\mathrm{benefit}\;\mathrm{criteria}\;C_{j},\\ {\left(\varepsilon_{ij}^{\left(e\right)}\right)}^{c} & \mathrm{for}\;\cos t\;\mathrm{criteria}\;C_{j},\; \end{array}\right.\\ e & = & 1,2,\ldots ,r,\;\;\;i=1,2,\ldots ,k,\;\;j=1,2,\ldots ,n, \end{array}$$
  where ${\left(\varepsilon_{ij}^{\left(e\right)}\right)}^{c}$ is complement of $\varepsilon_{ij}^{\left(e\right)}$, that is, ${\left(\varepsilon_{ij}^{\left(e\right)}\right)}^{c}=\left({\tilde{n}}_{\varepsilon_{ij}}^{\left(e\right)},\rho_{\varepsilon_{ij}}^{\left(e\right)}\right).$
Step 3. The weights of the criteria are calculated through proposed entropy measure in the following way. The entropy measure corresponding to each criterion is:
  $$\begin{array}{ccc} E\left(\varepsilon_{j}\right) & = & E\left(\varepsilon_{1j},\varepsilon_{2j},\ldots ,\varepsilon_{kj}\right),\;j=1,2,\ldots ,n\\ & = & \frac{-1}{\ln\left(m\right)}\sum_{i=1}^{m}\left(\rho_{h_{\varkappa }}\left(\ln\rho_{h_{\varkappa }}\right)\times p_{x}+{\tilde{n}}_{h_{\varkappa }}\left(\ln{\tilde{n}}_{h_{\varkappa }}\right)\times q_{x}\right) \end{array}$$
  Then,
  $$W\left(\varepsilon_{j}\right)=\frac{1-E\left(\varepsilon_{j}\right)}{\sum_{j=1}^{n}\left(1-E\left(\varepsilon_{j}\right)\right)}$$
  Thus weights of criteria are found as $W\left(\varepsilon_{j}\right)={\left[W\left(\varepsilon_{1}\right),\ldots ,W\left(\varepsilon_{n}\right)\right]}^{T}.$
Step 4. In this step, the TOPSIS method is applied on the calculated criteria weights to rank the alternatives corresponding to each decision matrix in the following way:
  Step 4a. Determine weighted NĐM^*e*^ through the evaluated criteria’s weights in the following way:
    $$\begin{array}{ccc} NĐM_{ij}^{\left(e\right)} & = & W\left(\varepsilon_{j}\right).\varepsilon_{ij}^{\left(e\right)}=\left(\begin{array}{c} \cup_{\gamma_{i}\in \rho_{\varepsilon_{ij}},p_{i}\in \rho_{\varepsilon_{ij}}}\sqrt{1-\Pi_{i=1}^{k}{\left(1-{\left(\gamma_{\varepsilon_{ij}^{\left(e\right)}}\right)}^{2}\right)}^{W\left(\varepsilon_{j}\right)}}/\Pi_{i=1}^{k}p_{\varepsilon_{ij}^{\left(e\right)}},\\ \cup_{\eta_{i}\in {\tilde{n}}_{\varepsilon_{ij}},q_{i}\in {\tilde{n}}_{\varepsilon_{ij}}}\Pi_{i=1}^{k}{\left(\eta_{\varepsilon_{ij}^{\left(e\right)}}\right)}^{W\left(\varepsilon_{j}\right)}/\Pi_{i=1}^{k}q_{\varepsilon_{ij}^{\left(e\right)}} \end{array}\right)\\ for\;\mathrm{all}\;e & = & 1,2,\ldots ,r. \end{array}$$
  Step 4b. Derive PIS^(*e*)^ and NIS^(*e*)^ for all weighted NĐM^(*e*)^, for all DM_*e*_(e=1,2,…,r) as follows [30]:
    $$\begin{array}{ccc} PIS^{\left(e\right)} & = & {\left\{PIS_{j}^{\left(e\right)}\right\}}_{r\times n}=\left\{\left(NĐM_{ij}^{\left(e\right)}\right):\max_{i}\left[s\left(NĐM_{ij}^{\left(e\right)}\right)\right]\right\}\\ \mathrm{for}\;j & = & 1,2,\ldots ,n \end{array}$$
    $$\begin{array}{ccc} NIS^{\left(e\right)} & = & {\left\{NIS_{j}^{\left(e\right)}\right\}}_{r\times n}=\left\{\left(NĐM_{ij}^{\left(e\right)}\right):\min_{i}\left[s\left(NĐM_{ij}^{\left(e\right)}\right)\right]\right\}\\ \mathrm{for}\;j & = & 1,2,\ldots ,n \end{array}$$
  Step 4c. Consider the evaluated criteria weights. Using weighted distance measure of weighted NĐM^(*e*)^ from $PIS^{\left(e\right)}$ and $NIS^{\left(e\right)}$ are denoted by ${\mathrm{EIS}}_{i}^{+}$ and ${\mathrm{EIS}}_{i}^{-},$ respectively, and are calculated corresponding to each DM_*e*_ in the following way:
    $$EIS_{i}^{+\left(e\right)}={\left(\sum_{j=1}^{n}\frac{1}{2n}W\left(\varepsilon_{j}\right)\left(\begin{array}{c} {\left|\frac{1}{M_{\varepsilon }}\sum_{i=1}^{M_{\varepsilon }}{\left(\gamma_{NĐM_{ij}^{\left(e\right)}}p_{NĐM_{ij}^{\left(e\right)}}\right)}^{2}-\frac{1}{M_{\vartheta }}\sum_{\tilde{ı}=1}^{M_{\vartheta }}{\left(\gamma_{PIS_{j}^{\left(e\right)}}p_{PIS_{j}^{\left(e\right)}}\right)}^{2}\right|}^{\zeta }+\\ {\left|\frac{1}{N_{\varepsilon }}\sum_{j=1}^{N_{\varepsilon }}{\left(\eta_{NĐM_{ij}^{\left(e\right)}}q_{NĐM_{ij}^{\left(e\right)}}\right)}^{2}-\frac{1}{N_{\vartheta }}\sum_{\hat{j}=1}^{N_{\vartheta }}{\left(\eta_{PIS_{j}^{\left(e\right)}}q_{PIS_{j}^{\left(e\right)}}\right)}^{2}\right|}^{\zeta } \end{array}\right)\right)}^{\frac{1}{\zeta }}$$
    $$\begin{array}{ccc} EIS_{i}^{-\left(e\right)} & = & {\left(\sum_{j=1}^{n}\frac{1}{2n}W\left(\varepsilon_{j}\right)\left(\begin{array}{c} {\left|\frac{1}{M_{\varepsilon }}\sum_{i=1}^{M_{\varepsilon }}{\left(\gamma_{NĐM_{ij}^{\left(e\right)}}p_{NĐM_{ij}^{\left(e\right)}}\right)}^{2}-\frac{1}{M_{\vartheta }}\sum_{\tilde{ı}=1}^{M_{\vartheta }}{\left(\gamma_{NIS_{j}^{\left(e\right)}}p_{NIS_{j}^{\left(e\right)}}\right)}^{2}\right|}^{\zeta }+\\ {\left|\frac{1}{N_{\varepsilon }}\sum_{j=1}^{N_{\varepsilon }}{\left(\eta_{NĐM_{ij}^{\left(e\right)}}q_{NĐM_{ij}^{\left(e\right)}}\right)}^{2}-\frac{1}{N_{\vartheta }}\sum_{\hat{j}=1}^{N_{\vartheta }}{\left(\eta_{NIS_{j}^{\left(e\right)}}q_{NIS_{j}^{\left(e\right)}}\right)}^{2}\right|}^{\zeta } \end{array}\right)\right)}^{\frac{1}{\zeta }}\\ \mathrm{for}\;i & = & 1,2,\ldots ,k \end{array}$$
  Step 4d. The revised closeness indices for all DM_*e*_ of the alternatives are determined in the following way:
    $$\overset{´}{R}CI_{i}^{e}=\frac{EIS_{i}^{-\left(e\right)}}{EIS_{i}^{-\left(e\right)}+EIS_{i}^{+\left(e\right)}}$$
Sep 5. Rank the alternative and choose the most desirable alternative having minimum distance.

**Figure 1:**
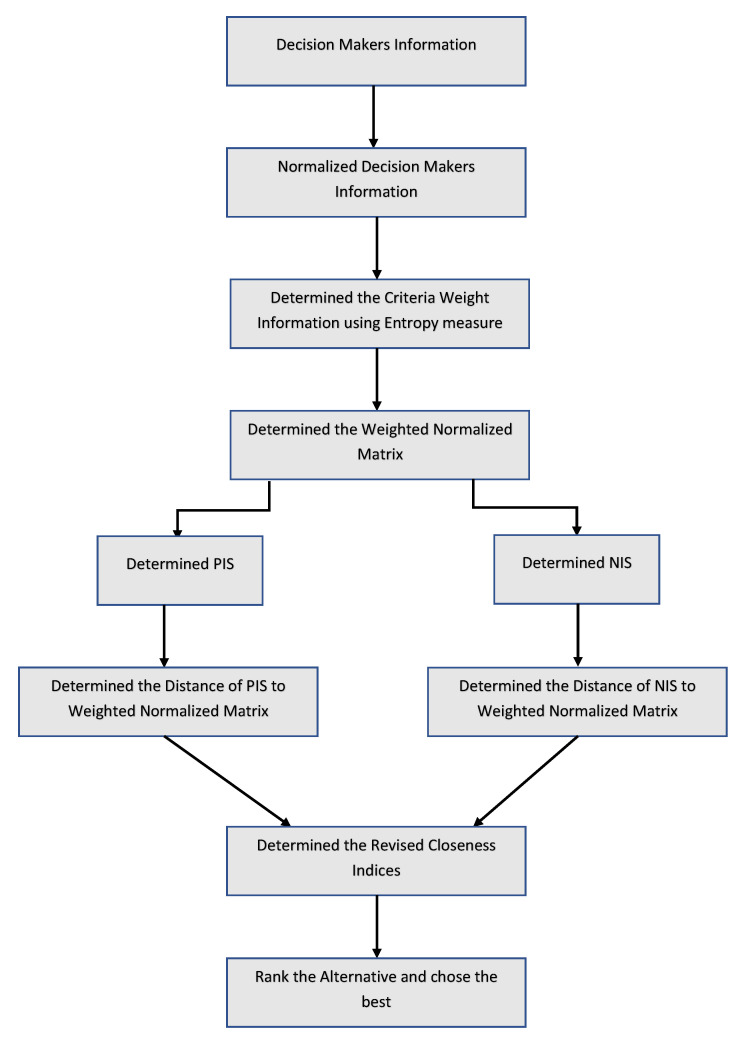
Flowchart of the proposed TOPSIS technique.

The flowchart of the proposed technique is given in Figure 1:

## 6. Numerical Example

In this section, we apply the developed decision making method to the fog-haze factor assessment problem to demonstrate the reliability and effectiveness of the proposed approach.

### Case Study

The rapid development of the economy and the acceleration of urbanization cause environmental problems, especially air pollution, which is increasingly prominent in Pakistan. Frequent fog-haze weather has affected people’s production and life, which attracts the Pakistani government’s great attention. Prime Minister Imran Khan called for more efforts to effectively deal with heavy pollution weather and strengthen research on the formation mechanism of fog-haze at Pakistan’s Two Sessions in 2019. Relevant departments of all provinces attach great importance to the prediction, early warning, prevention and control of fog-haze. The influence factors of fog-haze weather are complex and finding the most critical influence factor is important, which requires many experts to participate in the analysis of fog-haze factor.

Lahore, as the capital city of Punjab province of Pakistan, is increasingly polluted by fog-haze weather accompanied by the development of the economy in recent years, which is not conducive to Lahore’s economic development. In general, the main influence factors of fog-haze weather are PM10 concentration ${\bar{A}}_{1}$, PM2.5 concentration ${\bar{A}}_{2}$, geographical conditions ${\bar{A}}_{3}$, meteorological condition ${\bar{A}}_{4}$ and PM1.7 concentration ${\bar{A}}_{3}$. To assess the most critical influence factor of fog-haze weather under the list of criteria are ${\hat{C}}_{1},{\hat{C}}_{2}{\hat{C}}_{3}$ and ${\hat{C}}_{4}$, the city’s environmental protection department invites a group of related experts in the industry to evaluate the four influence factors. Because there exists too much uncertainty, the preference information given by the DMs is presented in the form of Pythagorean Probabilistic hesitant fuzzy information. To solve the MCDM problem by the developed methodology, the following calculations are achieved:

**Step** **1.** The decision maker information in the form of PyPHFNs is given in Table 1;
**Step** **2.** Information given by DMs are benefit criteria, so normalized values are carried out in Table 2, as follows
**Step 3.**  Compute the weight information for attributes/criteria using the proposed entropy measure of PyPHFNs as follows
  $$W=\left(W_{1}=0.110,W_{2}=0.387,W_{3}=0.207,W_{4}=0.296\right)$$
**Step 4a.**  Weighted normalized matrix is computed in Table 3, as follows.
**Step 4b.**  Positive and negative ideal solution are computed in Table 4, as follows
**Step 4c.**  Weighted distance measure of PIS and NIS to weighted normalized matrix are computed as
0.0780160.0452910.0751420.1195110.045982
and
0.0583340.0676310.0373030.0935020.066251
**Step 5.**  Ranking of the alternative is
0.4278240.5989160.3317440.4389510.590297
  Hence ${\bar{A}}_{3}$ alternative has the minimum distance, so it is the best alternative in the given list of alternatives.

## 7. Comparison Analysis

To verify the effectiveness of our proposed decision making method with intuitionistic hesitant fuzzy information, we make a comparative analysis between the proposed method and the previous method [18].

In [18] authors presented the similarity measures based on distance and cosine function; for comparison, the data set is shown in Table 5, as follows

Given weight vector is

$$W=\left(W_{1}=0.3,W_{2}=0.5,W_{3}=0.2\right)$$

Firstly, we calculate the weighted normalized decision matrix in Table 6, as follows

Positive and the negative ideal is computed in Table 7 as follows

Now, we compute the distance measure of PIS and NIS to weighted normalized matrix as follows
Distance of PIS to weighted normalized matrix.0.394990.223580.499420.23045
and
Distance of PIS to weighted normalized matrix.0.202180.121560.232590.20181

Finally, the ranking of the alternative is
0.338560.352210.317740.46686

Hence ${\bar{A}}_{3}$ alternative has the minimum distance, so it is the best alternative in the given list of alternatives. The comparison matrix is given in Table 8.

### Discussion

We use the proposed PyPHF-TOPSIS method to find the best choice among the given alternatives. According to our proposed method the result is similar and ${\bar{A}}_{3}$ is the best alternative. This shows the proposed method is also applicable to IHFSs and more effective and rational. The proposed method is an extension of existing methods. The proposed method can be considered as an alternative to solve more realistic decision making problems. The existing method is different from the proposed method. It is clear that existing IHF decision making process is not applicable for group decision making and it does not work if weights are unknown. However, the proposed PyPHF-TOPSIS method is applicable for group decision making and also when the weights of DMs and criteria are fully unknown. The proposed method also studies probabilistic information for each hesitant membership value and nonmembership value so there is no loss of probabilistic information.

Consequently, the proposed procedure is more precise, feasible and active to solve MCDM problems with fully unidentified information among DMs as well as criteria.

## 8. Conclusions

In this manuscript, we have proposed a novel hybrid concept of Pythagorean probabilistic hesitant fuzzy set to tackle the random vagueness, which is an enhanced version of fuzzy set, IFS, PyFS and PHFS in decision making real life problems. Firstly, we introduced some basic operational laws to deal with the PyPHFNs. Then, we developed new generalized distance measures for PyPHFSs. Furthermore, we proposed aggregation operator using PyPHF information. The main benefit of the proposed operator is that it provides the probabilistic information to all Pythagorean hesitant positive and negative degrees which provide a good tool for the DM to make a decision efficiently. This developed operator has been applied to the MCDM problem using extended TOPSIS technique. To show the effectiveness and applicability of the proposed extended TOPSIS approach under PyPHF, information is illustrated by numerical example. A comparison analysis is also considered to authenticate the applicability of our proposed method in practical MADM problems. The main advantages of this manuscript are as follows:

(1) the TOPSIS method is generalized by PyPHFS with unidentified weight information
(2) the PyPHF-TOPSIS method is proposed to solve the PyPHF-MCGDM problems with entropy weight
(3) a case study for car allotment site selection is provided to show the proposed approach
(4) our proposed PyPHF-TOPSIS method makes the distance similarity degree from the positive and negative ideal solution with entropy weight prominent and
(5) in this study, the TOPSIS method, which may be also a significant MADM or MAGDM method, has been considered to challenge ambiguous decision making problems.

In future research, considering the superiority of PyPHFSs, we can extend them to other methods, such as TODIM method, MABAC method, MULTIMOORA method, ORESTE method and so forth. In addition, we can propose some new weighting models to obtain attribute weights. In order to be able to apply these new methods better to actual decision making, we can use them to solve some medical diagnosis and risk investment problems.

## Figures and Tables

**Table 1:** Collective information of decision makers.

$\varepsilon^{\left(e\right)}$	${\hat{C}}_{1}$	${\hat{C}}_{2}$	${\hat{C}}_{3}$	${\hat{C}}_{4}$
${\bar{A}}_{1}$	$\begin{array}{c} \left(0.4/0.6,0.6/0.4\right)\\ \left(0.2/0.6,0.3/0.4\right) \end{array}$	$\begin{array}{c} \left(0.1/1\right)\\ \left(0.6/0.6,0.8/0.4\right) \end{array}$	$\begin{array}{c} \left(0.1/0.9,0.3/0.1\right)\\ \left(0.6/0.7,0.7/0.3\right) \end{array}$	$\begin{array}{c} \left(0.6/0.4,0.5/0.6\right)\\ \left(0.1/1\right) \end{array}$
${\bar{A}}_{2}$	$\begin{array}{c} \left(0.1/0.3,0.3/0.7\right)\\ \left(0.5/1\right) \end{array}$	$\begin{array}{c} \left(0.5/0.4,0.6/0.6\right)\\ \left(0.3/0.7,0.4/0.3\right) \end{array}$	$\begin{array}{c} \left(0.4/1\right)\\ \left(0.3/0.6,0.2/0.4\right) \end{array}$	$\begin{array}{c} \left(0.3/0.2,0.2/0.8\right)\\ \left(0.6/0.5,0.3/0.5\right) \end{array}$
${\bar{A}}_{3}$	$\begin{array}{c} \left(0.5/0.5,0.7/0.5\right)\\ \left(0.1/0.5,0.2/0.5\right) \end{array}$	$\begin{array}{c} \left(0.1/1\right)\\ \left(0.3/0.4,0.4/0.6\right) \end{array}$	$\begin{array}{c} \left(0.5/0.5,0.6/0.5\right)\\ \left(0.3/0.9,0.1/0.1\right) \end{array}$	$\begin{array}{c} \left(0.1/0.1,0.1/0.9\right)\\ \left(0.7/1\right) \end{array}$
${\bar{A}}_{4}$	$\begin{array}{c} \left(0.4/0.4,0.6/0.6\right)\\ \left(0.3/1\right) \end{array}$	$\begin{array}{c} \left(0.7/0.5,0.9/0.5\right)\\ \left(0.1/0.5,0.1/0.5\right) \end{array}$	$\begin{array}{c} \left(0.2/1\right)\\ \left(0.3/0.2,0.6/0.8\right) \end{array}$	$\begin{array}{c} \left(0.4/0.5,0.6/0.5\right)\\ \left(0.3/0.9,0.2/0.1\right) \end{array}$
${\bar{A}}_{5}$	$\begin{array}{c} \left(0.3/0.3,0.6/0.7\right)\\ \left(0.2/0.6,0.3/0.4\right) \end{array}$	$\begin{array}{c} \left(0.3/1\right)\\ \left(0.6/0.8,0.3/0.2\right) \end{array}$	$\begin{array}{c} \left(0.7/0.4,0.6/0.6\right)\\ \left(0.1/0.1,0.3/0.9\right) \end{array}$	$\begin{array}{c} \left(0.7/0.3,0.4/0.7\right)\\ \left(0.2/1\right) \end{array}$

**Table 2:** Normalized collective information of decision makers.

$\varepsilon^{\left(e\right)}$	${\hat{C}}_{1}$	${\hat{C}}_{2}$	${\hat{C}}_{3}$	${\hat{C}}_{4}$
${\bar{A}}_{1}$	$\begin{array}{c} \left(0.4/0.6,0.6/0.4\right)\\ \left(0.2/0.6,0.3/0.4\right) \end{array}$	$\begin{array}{c} \left(0.1/1\right)\\ \left(0.6/0.6,0.8/0.4\right) \end{array}$	$\begin{array}{c} \left(0.1/0.9,0.3/0.1\right)\\ \left(0.6/0.7,0.7/0.3\right) \end{array}$	$\begin{array}{c} \left(0.6/0.4,0.5/0.6\right)\\ \left(0.1/1\right) \end{array}$
${\bar{A}}_{2}$	$\begin{array}{c} \left(0.1/0.3,0.3/0.7\right)\\ \left(0.5/1\right) \end{array}$	$\begin{array}{c} \left(0.5/0.4,0.6/0.6\right)\\ \left(0.3/0.7,0.4/0.3\right) \end{array}$	$\begin{array}{c} \left(0.4/1\right)\\ \left(0.3/0.6,0.2/0.4\right) \end{array}$	$\begin{array}{c} \left(0.3/0.2,0.2/0.8\right)\\ \left(0.6/0.5,0.3/0.5\right) \end{array}$
${\bar{A}}_{3}$	$\begin{array}{c} \left(0.5/0.5,0.7/0.5\right)\\ \left(0.1/0.5,0.2/0.5\right) \end{array}$	$\begin{array}{c} \left(0.1/1\right)\\ \left(0.3/0.4,0.4/0.6\right) \end{array}$	$\begin{array}{c} \left(0.5/0.5,0.6/0.5\right)\\ \left(0.3/0.9,0.1/0.1\right) \end{array}$	$\begin{array}{c} \left(0.1/0.1,0.1/0.9\right)\\ \left(0.7/1\right) \end{array}$
${\bar{A}}_{4}$	$\begin{array}{c} \left(0.4/0.4,0.6/0.6\right)\\ \left(0.3/1\right) \end{array}$	$\begin{array}{c} \left(0.7/0.5,0.9/0.5\right)\\ \left(0.1/0.5,0.1/0.5\right) \end{array}$	$\begin{array}{c} \left(0.2/1\right)\\ \left(0.3/0.2,0.6/0.8\right) \end{array}$	$\begin{array}{c} \left(0.4/0.5,0.6/0.5\right)\\ \left(0.3/0.9,0.2/0.1\right) \end{array}$
${\bar{A}}_{5}$	$\begin{array}{c} \left(0.3/0.3,0.6/0.7\right)\\ \left(0.2/0.6,0.3/0.4\right) \end{array}$	$\begin{array}{c} \left(0.3/1\right)\\ \left(0.6/0.8,0.3/0.2\right) \end{array}$	$\begin{array}{c} \left(0.7/0.4,0.6/0.6\right)\\ \left(0.1/0.1,0.3/0.9\right) \end{array}$	$\begin{array}{c} \left(0.7/0.3,0.4/0.7\right)\\ \left(0.2/1\right) \end{array}$

**Table 3:** (**a**) Weighted normalized matrix. (**b**) Weighted normalized matrix.

(**a**)		
$\varepsilon^{\left(e\right)}$	${\hat{C}}_{1}$	${\hat{C}}_{2}$
${\bar{A}}_{1}$	$\begin{array}{c} \left(0.137826/0.6,0.218875/0.4\right)\\ \left(0.837748/0.6,0.875958/0.4\right) \end{array}$	$\begin{array}{c} \left(0.062305/1\right)\\ \left(0.820625/0.6,0.917267/0.4\right) \end{array}$
${\bar{A}}_{2}$	$\begin{array}{c} \left(0.03324/0.3,0.10159/0.7\right)\\ \left(0.926588/1\right) \end{array}$	$\begin{array}{c} \left(0.324591/0.4,0.398273/0.6\right)\\ \left(0.627547/0.7,0.701451/0.3\right) \end{array}$
${\bar{A}}_{3}$	$\begin{array}{c} \left(0.176492/0.5,0.267192/0.5\right)\\ \left(0.776247/0.5,0.837748/0.5\right) \end{array}$	$\begin{array}{c} \left(0.062305/1\right)\\ \left(0.627547/0.4,0.701451/0.6\right) \end{array}$
${\bar{A}}_{4}$	$\begin{array}{c} \left(0.137826/0.4,0.218875/0.6\right)\\ \left(0.875958/1\right) \end{array}$	$\begin{array}{c} \left(0.478956/0.5,0.688572/0.5\right)\\ \left(0.410204/0.5,0.410204/0.5\right) \end{array}$
${\bar{A}}_{5}$	$\begin{array}{c} \left(0.10159/0.3,0.218875/0.7\right)\\ \left(0.837748/0.6,0.875958/0.4\right) \end{array}$	$\begin{array}{c} \left(0.189315/1\right)\\ \left(0.820625/0.8,0.627547/0.2\right) \end{array}$
(**b**)		
$\varepsilon^{\left(e\right)}$	${\hat{C}}_{3}$	${\hat{C}}_{4}$
${\bar{A}}_{1}$	$\begin{array}{c} \left(0.045588/0.9,0.139043/0.1\right)\\ \left(0.899658/0.7,0.928828/0.3\right) \end{array}$	$\begin{array}{c} \left(0.351778/0.4,0.285708/0.6\right)\\ \left(0.505825/1\right) \end{array}$
${\bar{A}}_{2}$	$\begin{array}{c} \left(0.188275/1\right)\\ \left(0.779407/0.6,0.71666/0.4\right) \end{array}$	$\begin{array}{c} \left(0.165921/0.2,0.109593/0.8\right)\\ \left(0.859672/0.5,0.700209/0.5\right) \end{array}$
${\bar{A}}_{3}$	$\begin{array}{c} \left(0.240441/0.5,0.297057/0.5\right)\\ \left(0.779407/0.9,0.620869/0.1\right) \end{array}$	$\begin{array}{c} \left(0.054502/0.1,0.054502/0.9\right)\\ \left(0.899806/1\right) \end{array}$
${\bar{A}}_{4}$	$\begin{array}{c} \left(0.091731/1\right)\\ \left(0.779407/0.2,0.899658/0.8\right) \end{array}$	$\begin{array}{c} \left(0.224275/0.5,0.351778/0.5\right)\\ \left(0.700209/0.9,0.621019/0.1\right) \end{array}$
${\bar{A}}_{5}$	$\begin{array}{c} \left(0.3607/0.4,0.297057/0.6\right)\\ \left(0.620869/0.1,0.72/0.9\right) \end{array}$	$\begin{array}{c} \left(0.425093/0.3,0.224275/0.7\right)\\ \left(0.621019/1\right) \end{array}$

**Table 4:** (**a**) Positive ideal solution (PIS). (**b**) Negative ideal solution (NIS).

(**a**)			
(0.176/0.5,0.267/0.5)	(0.479/0.5,0.689/0.5)	(0.36/0.4,0.297/0.6)	(0.352/0.4,0.286/0.6)
(0.776/0.5,0.837/0.5)	(0.41/0.5,0.41/0.5)	(0.621/0.1,0.779/0.9)	(0.506/1)
(**b**)			
(0.033/0.3,0.102/0.7)	(0.062/1)	(0.046/0.9,0.139/0.1)	(0.055/0.1,0.055/0.9)
(0.927/1)	(0.821/0.6,0.917/0.4)	(0.899/0.7,0.929/0.3)	(0.899/1)

**Table 5:** Collective information of decision makers.

$\varepsilon^{\left(e\right)}$	${\hat{C}}_{1}$	${\hat{C}}_{2}$	${\hat{C}}_{3}$
${\bar{A}}_{1}$	$\left(0.8,0.6,0.5\right)\left(0.1,0.2,0.4\right)$	$\left(0.6,0.4\right)\left(0.4,0.5\right)$	$\left(0.9,0.6,0.5\right)\left(0.1,0.3,0.4\right)$
${\bar{A}}_{2}$	$\left(0.7,0.5,0.4\right)\left(0.3,0.4,0.4\right)$	$\left(0.6,0.4,0.2\right)\left(0.3,0.3,0.5\right)$	$\left(0.8,0.4\right)\left(0.2,0.3\right)$
${\bar{A}}_{3}$	$\left(0.7,0.5,0.3\right)\left(0.2,0.2,0.6\right)$	$\left(0.9,0.7,0.5\right)\left(0.1,0.2,0.4\right)$	$\left(0.5,0.4,0.3\right)\left(0.4,0.6,0.6\right)$
${\bar{A}}_{4}$	$\left(0.5,0.4,0.3\right)\left(0.3,0.5,0.6\right)$	$\left(0.7,0.5\right)\left(0.2,0.3\right)$	$\left(0.8,0.7\right)\left(0.1,0.3\right)$

**Table 6:** (**a**) Weighted normalized matrix. (**b**) Weighted normalized matrix.

(**a**)		
$\varepsilon^{\left(e\right)}$	${\hat{C}}_{1}$	${\hat{C}}_{2}$
${\bar{A}}_{1}$	$\left(0.51,0.35,0.29\right)\left(0.50,0.61,0.76\right)$	$\left(0.45,0.29\right)\left(0.63,0.70\right)$
${\bar{A}}_{2}$	$\left(0.43,0.29,0.23\right)\left(0.70,0.76,0.76\right)$	$\left(0.45,0.29,0.14\right)\left(0.55,0.55,0.70\right)$
${\bar{A}}_{3}$	$\left(0.43,0.29,0.17\right)\left(0.62,0.62,0.86\right)$	$\left(0.75,0.53,0.37\right)\left(0.32,0.45,0.63\right)$
${\bar{A}}_{4}$	$\left(0.29,0.23,0.17\right)\left(0.70,0.81,0.86\right)$	$\left(0.53,0.37\right)\left(0.45,0.55\right)$
(**b**)		
$\varepsilon^{\left(e\right)}$	${\hat{C}}_{3}$	
${\bar{A}}_{1}$	$\left(0.53,0.29,0.24\right)\left(0.63,0.79,0.83\right)$	
${\bar{A}}_{2}$	$\left(0.43,0.19\right)\left(0.72,0.79\right)$	
${\bar{A}}_{3}$	$\left(0.24,0.19,0.14\right)\left(0.83,0.90,0.90\right)$	
${\bar{A}}_{4}$	$\left(0.43,0.35\right)\left(0.63,0.79\right)$	

**Table 7:** (**a**) PIS; (**b**) NIS.

(**a**)		
$\left(0.51,0.35,0.29\right)\left(0.50,0.61,0.76\right)$	$\left(0.75,0.53,0.37\right)\left(0.32,0.45,0.63\right)$	$\left(0.43,0.35\right)\left(0.63,0.79\right)$
(**b**)		
$\left(0.29,0.23,0.17\right)\left(0.70,0.81,0.86\right)$	$\left(0.45,0.29,0.14\right)\left(0.55,0.55,0.70\right)$	$\left(0.24,0.19,0.14\right)\left(0.83,0.90,0.90\right)$

**Table 8:** Comparison matrix.

	Revised Closeness Indices	Ranking
Existing Technique [18]	0.338560.352210.317740.46686	${\bar{A}}_{3}>{\bar{A}}_{1}>{\bar{A}}_{2}>{\bar{A}}_{4}$
Propose Technique	0.42780.59890.33170.43890.5902	${\bar{A}}_{3}>{\bar{A}}_{1}>{\bar{A}}_{4}>{\bar{A}}_{5}>{\bar{A}}_{2}$
